# Chronic Recurrent Esophageal Diverticulitis - A Rare Entity

**DOI:** 10.4021/gr522w

**Published:** 2013-03-09

**Authors:** Ashish Manne, Ioana Smith, Jeremy Hatchett, Jeffrey Juneau, Sudha Kodali, Talha A. Malik, Fred H. Weber

**Affiliations:** a Division of Gastroenterology/Hepatology, University of Alabama at Birmingham, Birmingham, Alabama, USA

**Keywords:** Esophageal diverticulitis, Upper endoscopy, Barium swallow

## Abstract

In this report, we seek to shed light on a 44-year-old Caucasian male with a known history of an esophageal diverticulum, who was transferred to our facility after an upper endoscopy at an outside hospital suggested a purulent discharge emanating from the mouth of a mid-esophageal diverticulum. A barium swallow done at the outside institution had reportedly demonstrated an 8 cm long barium collection parallel to and anterolateral to the mid-and distal esophagus which terminated several centimeters proximal to the gastroesophageal junction. At our facility, antibiotics (piperacillin/tazobactam) were continued, and a double-contrast esophagram was performed. The presence of an unusual mid-esophageal diverticulum was confirmed. He clinically improved after a 3-day course of intravenous broad-spectrum antibiotics. No surgical or endoscopic repair was elected as the patient opted for continued medical management. While esophageal diverticula are not rare in humans, to our knowledge, this is the first report of development of esophageal diverticulitis in humans. We believe that antibiotic coverage in addition to dietary restriction is the logical mainstay of acute therapy. Optimal antibiotic coverage should likely include oral flora aerobes and anaerobes. Once symptoms resolve, diverticula may be managed expectantly.

## Introduction

While esophageal diverticula are not rare in humans, development of esophageal diverticulitis in humans has not been reported in the literature. In this report, we aim to describe a 44-year-old Caucasian male who was confirmed to have esophageal diverticulitis at our facility and underwent successful management. After description of the case, we shed light on pathogenesis and clinical presentation of this condition followed by a brief discourse on management options.

## Case Report

A 44-year-old Caucasian male with a known history of an esophageal diverticulum, gastroesophageal reflux (GER), and dysphagia since the 1980s was transferred to our hospital after an upper endoscopy at an outside facility. Upper endoscopy there suggested a purulent discharge emanating from the mouth of a mid-esophageal diverticulum. Of note, he had presented to the outside facility with moderate to severe chest pain radiating to the back accompanied by fever to 38.9 °C and leukocytosis. A barium swallow revealed an 8 cm long barium collection parallel to and anterolateral to the mid-and distal esophagus which terminated several centimeters proximal to the gastroesophageal junction. CT imaging demonstrated mild circumferential distal esophageal wall thickening. He described a twenty five year history of periodic, stereotypic episodes of prodromal pleuritic chest pain followed by fever to 38.9 °C, and sustained chest and epigastric pain. Such episodes had been occurring 1 - 2 times yearly and would resolve over 3 - 5 days with oral antibiotics. More severe episodes had been requiring hospital admission for IV antibiotics. Long-standing intermittent solid food dysphagia had required him to drink liquids when ingesting solid foods.

**Figure 1:**
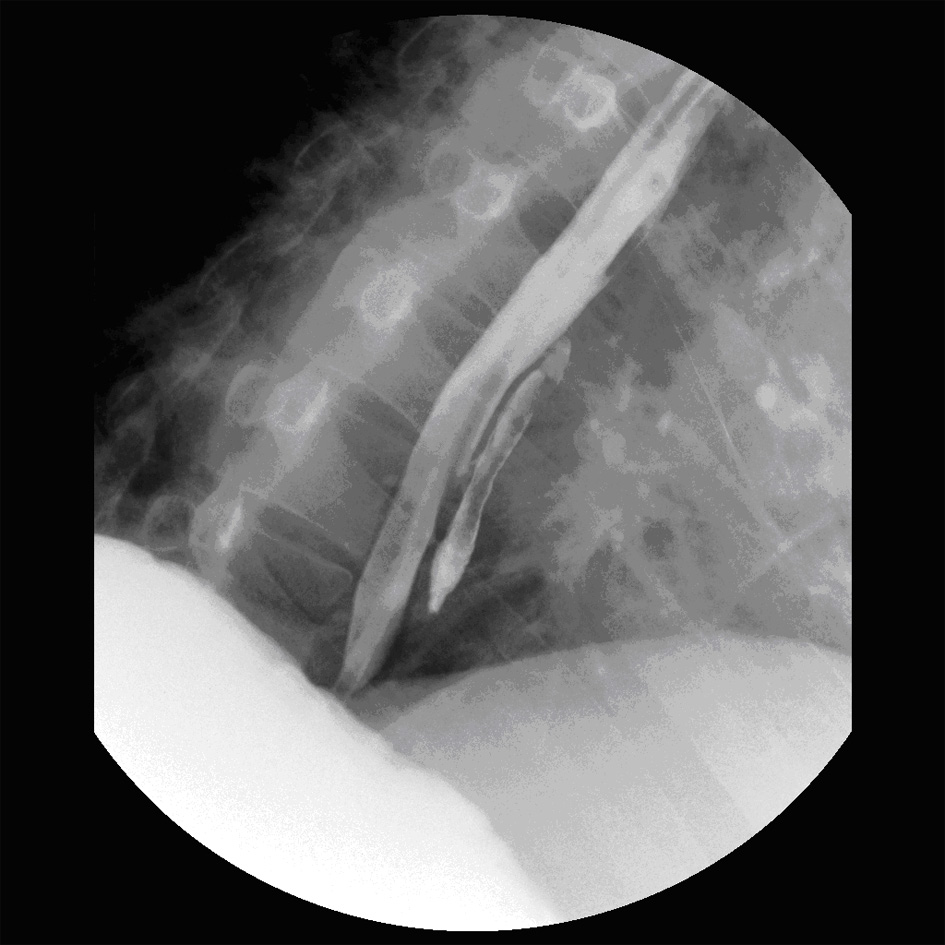
Double contrast esophagram demonstrating a mid-esophageal diverticulum.

At our facility, antibiotics (piperacillin/tazobactam) were continued, and a double-contrast esophagram was performed. The presence of an unusual mid-esophageal diverticulum was confirmed (Fig. 1). Esophageal manometry was normal. He clinically improved after a 3-day course of intravenous broad-spectrum antibiotics. No surgical or endoscopic repair was elected as the patient opted for continued medical management.

## Discussion

Esophageal diverticula are not rare but to our knowledge, this is the first report of esophageal diverticulitis in humans to be reported in the literature. However, an esophageal diverticulum associated with an *Aerococcus viridians* infection in a loggerhead sea turtle (*Caretta caretta*) has been described [1].

Esophageal diverticula can be congenital or acquired [2]. Acquired esophageal diverticula include cricopharyngeal muscle weakness-related *Zenker’s diverticulum* [3], terminally located epiphrenic diverticula also known as pulsion diverticula usually associated with motility disorders [4], and those associated with pathological conditions like tuberculosis where the tractional force of lymphadenitis results in midesophageal or traction diverticula [5].

As esophageal diverticulitis has not been reported in humans, the mechanism of its development in our patient is speculative. Ingested food particles may lodge in the distal recesses or mouth of the diverticulum resulting in stasis. In regard to this case, the predisposing factor may have been the very unusual anatomy of the diverticulum with very long length and small entrance likely predisposing to stasis.

Signs and symptoms may include severe inspiratory chest pain, fever with chills and rigors, dysphagia, odynophagia, and leukocytosis, along with regurgitation, choking, nausea, and vomiting.

Differential diagnoses include angina/myocardial infarction, pleuritis/pericarditis, gastroesophageal reflux disease, motility disorders (achalasia, diffuse esophageal spasm, nutcracker esophagus, and scleroderma), paraesophageal hernia with infection, esophageal stricture, and esophageal cancer. Diagnosis of esophageal diverticulitis may be made with the combination of clinical assessment, CBC with differential testing, barium swallow, CT scan and upper endoscopy.

Antibiotics and dietary restriction are the logical mainstay of acute therapy. Optimal antibiotic coverage should likely include oral flora aerobes and anaerobes; it is unclear whether gram negative rod coverage is necessary. Once symptoms resolve, diverticula may be managed expectantly. Recurrent or severe episodes may warrant endoscopic or surgical (diverticulectomy, myotomy, and possible fundoplication) management options as have been described for such symptomatic lesions outside of an acute diverticulitis scenario [6].
